# Toward Timely Data for Cancer Research: Assessment and Reengineering of the Cancer Reporting Process

**DOI:** 10.2196/cancer.7515

**Published:** 2018-03-01

**Authors:** Abdulrahman M Jabour, Brian E Dixon, Josette F Jones, David A Haggstrom

**Affiliations:** ^1^ Health Informatics Department Faculty of Public Health and Tropical Medicine Jazan University Jazan Saudi Arabia; ^2^ Department of BioHealth Informatics School of Informatics and Computing Indiana University & Purdue University Indianapolis Indianapolis, IN United States; ^3^ Center for Biomedical Informatics Regenstrief Institute Indianapolis, IN United States; ^4^ Department of Epidemiology Richard M Fairbanks School of Public Health Indiana University Indianapolis, IN United States; ^5^ Health Services Research and Development Center for Health Information and Communication Roudebush Veterans Affairs Medical Center Indianapolis, IN United States; ^6^ Division of General Internal Medicine Department of Medicine Indiana University Indianapolis, IN United States; ^7^ Center for Health Services Research Regenstrief Institute Indianapolis, IN United States

**Keywords:** neoplasms, registries, SEER program, workflow, computer simulation, data collection, epidemiological monitoring

## Abstract

**Background:**

Cancer registries systematically collect cancer-related data to support cancer surveillance activities. However, cancer data are often unavailable for months to years after diagnosis, limiting its utility.

**Objective:**

The objective of this study was to identify the barriers to rapid cancer reporting and identify ways to shorten the turnaround time.

**Methods:**

Certified cancer registrars reporting to the Indiana State Department of Health cancer registry participated in a semistructured interview. Registrars were asked to describe the reporting process, estimate the duration of each step, and identify any barriers that may impact the reporting speed. Qualitative data analysis was performed with the intent of generating recommendations for workflow redesign. The existing and redesigned workflows were simulated for comparison.

**Results:**

Barriers to rapid reporting included access to medical records from multiple facilities and the waiting period from diagnosis to treatment. The redesigned workflow focused on facilitating data sharing between registrars and applying a more efficient queuing technique while registrars await the delivery of treatment. The simulation results demonstrated that our recommendations to reduce the waiting period and share information could potentially improve the average reporting speed by 87 days.

**Conclusions:**

Knowing the time elapsing at each step within the reporting process helps in prioritizing the needs and estimating the impact of future interventions. Where some previous studies focused on automating some of the cancer reporting activities, we anticipate much shorter reporting by leveraging health information technologies to target this waiting period.

## Introduction

### Data Quality in Cancer Registries

Despite multiple reports from the National Academy of Medicine (NAM; formerly Institute of Medicine) dating back to 1999, achieving higher quality cancer care remains a challenge [1]. In its latest report, the NAM recommended leveraging health information technologies to create a Rapid Learning System in which the latest evidence and knowledge regarding cancer case outcomes is fed back into cancer care delivery processes and treatments [1]. One specific recommendation is to leverage cancer registries together with electronic health record (EHR) systems to enable timely capture and reporting of data [2,3]. Current approaches can take more than a year after diagnosis before data are available at state-based cancer registries for wider use [4]. Despite the rich data available in cancer registries, the lengthy reporting time poses a major barrier to using these data for real-time, actionable outcome and quality reports [1-5].

### Understanding Cancer Reporting Process

There is limited evidence on the reporting process, the barriers to more rapid reporting, or precisely how EHR systems might be used to improve timeliness. Existing studies largely examine factors associated with the timeliness of cancer data [6-10]. For example, a study in the first group by Gagen and Cress investigated the association between reporting delays and gender, race, type of reporting facility, cancer site, and stage at diagnosis [10]. Of these factors, the type of reporting facility (eg, hospital, physician’s office, and laboratory) was associated with reporting time; cases reported by hospitals had shorter reporting times compared with those reported by physician offices or laboratory centers [10]. At least one prior study focused on EHR systems’ impact on cancer registries. Among a convenience sample of cancer registrars, Houser et al asked attendees at a conference whether they used EHR systems to access data, as well as their perceptions of the benefits and challenges associated with EHR usage [11]. Although this study found that EHR systems were being used and viewed favorably by the majority of sampled conference attendees, the study did not provide detailed insights into the sequence of reporting tasks or the workflow efficiency.

While providing an important foundation, prior research has not described the precise challenges associated with the sequence of steps involved in the cancer reporting process or potential solutions to address specific challenges. Cancer reporting is complex, labor-intensive, and typically performed by certified cancer registrars referred to as certified tumor registrars (CTRs). Registrars are data information specialists who capture the complete medical history for cancer patients including diagnosis, treatment, and health status and then report this information to cancer registries [12]. Cancer registrars must compile patient data from various sources, analyze these data, and enter the data into a complete, uniform abstract. These abstracts must then be transmitted along a reporting chain spanning hospitals, state health agencies, and the national Centers for Disease Control and Prevention (CDC). Reporting turnaround time is largely dependent upon the activities performed at the hospital level by cancer registrars such as data searching, collection, and abstraction. Reduction in the reporting time cannot be achieved without a comprehensive understanding of the reporting workflow and challenges faced by CTRs at the hospital level. To address similar challenges, many studies have shown the value of workflow evaluation in navigating the complexity of health care systems. Workflow evaluation has been used in health care settings such as emergency departments, primary care, pharmacy, and radiology departments [13-17]. These studies commonly utilize some combination of field observations and in-depth interviews. Although field observations can reveal details that users might overlook, in-depth interviews can provide a deeper understanding of the processes and tasks involved, such as task descriptions, alternative routes, the rationale for given choices, and the difficulties encountered.

To investigate the reporting process and identify barriers to timely reporting, we conducted key informant interviews with CTRs across the state of Indiana. Insights from the interviews were translated into input for simulations of the reporting process to explore ways that the reporting time could be decreased. In addition, the study explored ways in which EHR systems and health information exchange (HIE) could be leveraged to improve cancer reporting data timeliness.

## Methods

### Study Design

To better understand the complex processes involved in cancer case reporting, we conducted a multi-phase study. First, we interviewed cancer registrars to identify barriers to timely reporting and developed a model of current reporting processes. Second, we developed computer simulation models to represent the current state and a potential, redesigned future state. The outputs of the two simulations were compared to determine the impact of health information technology innovations, including the use of EHR systems that might be implemented to increase the speed of cancer case reporting processes. The study was approved by the institutional review board at Indiana University.

### System and Scope

In the United States, all 50 individual states have programs for cancer surveillance, involving the routine collection and compilation of specified clinical and demographic information about every newly diagnosed, reportable cancer [18,19]. Hospitals report cancer cases to state-level registries operated by public health authorities, which in turn report to nationwide registries to enable population-based analysis. Cases received by state registries are reported to the Surveillance, Epidemiology, and End Results program operated by the National Cancer Institute and/or the National Program of Cancer Registries operated by the CDC [18-23].

In this study, we examine the US state-level cancer reporting process by interviewing CTRs who report on behalf of hospitals to the Indiana State Department of Health (ISDH) cancer registry. The ISDH cancer registry collects information related to tumor cases diagnosed or treated within the State of Indiana as required by state law or federal regulations [22].

The information obtained by the ISDH cancer registry includes demographic, treatment, and diagnostic data that are used for a wide range of activities, including epidemiologic studies of cancer causes and outcomes that can inform public health policies [22].

### Study Participants and Recruitment

In this study, recruitment was limited to CTRs who report case information to the ISDH cancer registry. Participants were invited to participate in either face-to-face or telephone interviews. Participants were identified through hospital staff directories and the Indiana Cancer Registrars Association. When recruiting participants, we directly contacted registrars reporting for larger hospitals (with 300 beds or more) within Indianapolis. To include registrars reporting for smaller hospitals and individual facilities, registrars from the Indiana Cancer Registrars Association directory were invited via email. Nonrespondents were reminded 2 weeks after the initial invitation. Snowball sampling, wherein initial contacts identify other individuals who may have insight into the topics of interest, was also used to expand the number of participants.

Recruitment occurred over a 5-month period between the end of March and August 2015. The recruitment process was concurrent with the development of the workflow and simulation models to validate model assumptions and compare the simulation output with the real system. Participants were identified and approached until saturation was achieved [24], that is, until no new themes or ideas were found. Upon completion of the interview, participants were thanked with a US $20 gift card.

### Interview Guide

Interviews were semistructured and task-oriented. The interview guide was developed to investigate the following areas: (1) understanding the workflow of cancer reporting, (2) estimating the time spent on each phase within the process, and (3) identifying the barriers to rapid reporting (Multimedia Appendix 1). Follow-up questions were asked for clarification and to confirm the representation of the developed model. Probing questions were asked to investigate additional information such as decision-making processes and alternative processes. For example, participants were asked to estimate the time required to complete abstraction of case information from the patient’s record. Later, they were asked if there are any types of information that take longer than others to abstract, and if so, how often they encounter these data types. In addition to describing the present state of cancer registry reporting, participants were asked to freely envision and describe optimal cancer reporting mechanisms, enabling them to transcend concerns for current resources or structural limitations [25].

### Analysis

Interview data were analyzed using a grounded theory approach [26-28]. We employed the following analysis steps: open coding, axial coding, and selective coding [26]. During the open coding step, keywords, phrases, and ideas were extracted to develop concepts and subcategories. Examples of these subcategories included barriers, facilitators, duration of each subtask, and reporting sources. During the axial coding, we grouped the concepts and subcategories into similar categories and considered the relationships among them. One relationship included the reporting step in which a barrier was encountered and a facilitator was used to overcome the barrier. For example, when CTRs reported difficulties accessing information from external hospitals, we examined whether the difficulties were encountered during the case finding or the abstracting phase. We further examined whether a barrier was encountered for all cancer types or a particular type of cancer, as well as whether a barrier was reported by CTRs from all hospitals or a subgroup of hospitals (eg, large hospitals). During selective coding, we used the derived categories to form higher-level themes. The analysis was performed using NVivo 10 (developed by QSR international).

### Flowchart and Simulation Development

**Figure 1:**
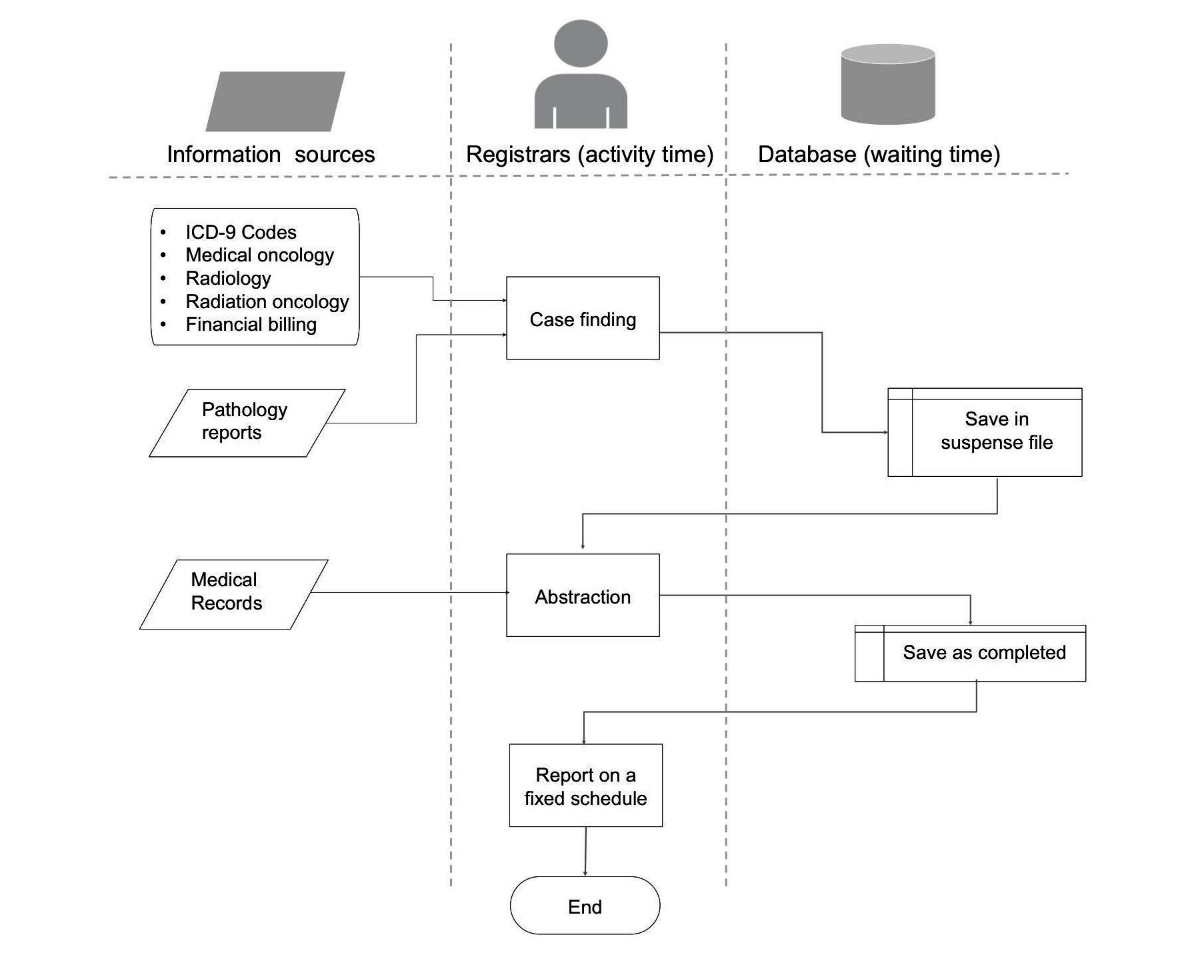
Cancer reporting flowchart for the existing workflow. ICD: International Classification of Diseases.

**Figure 2:**
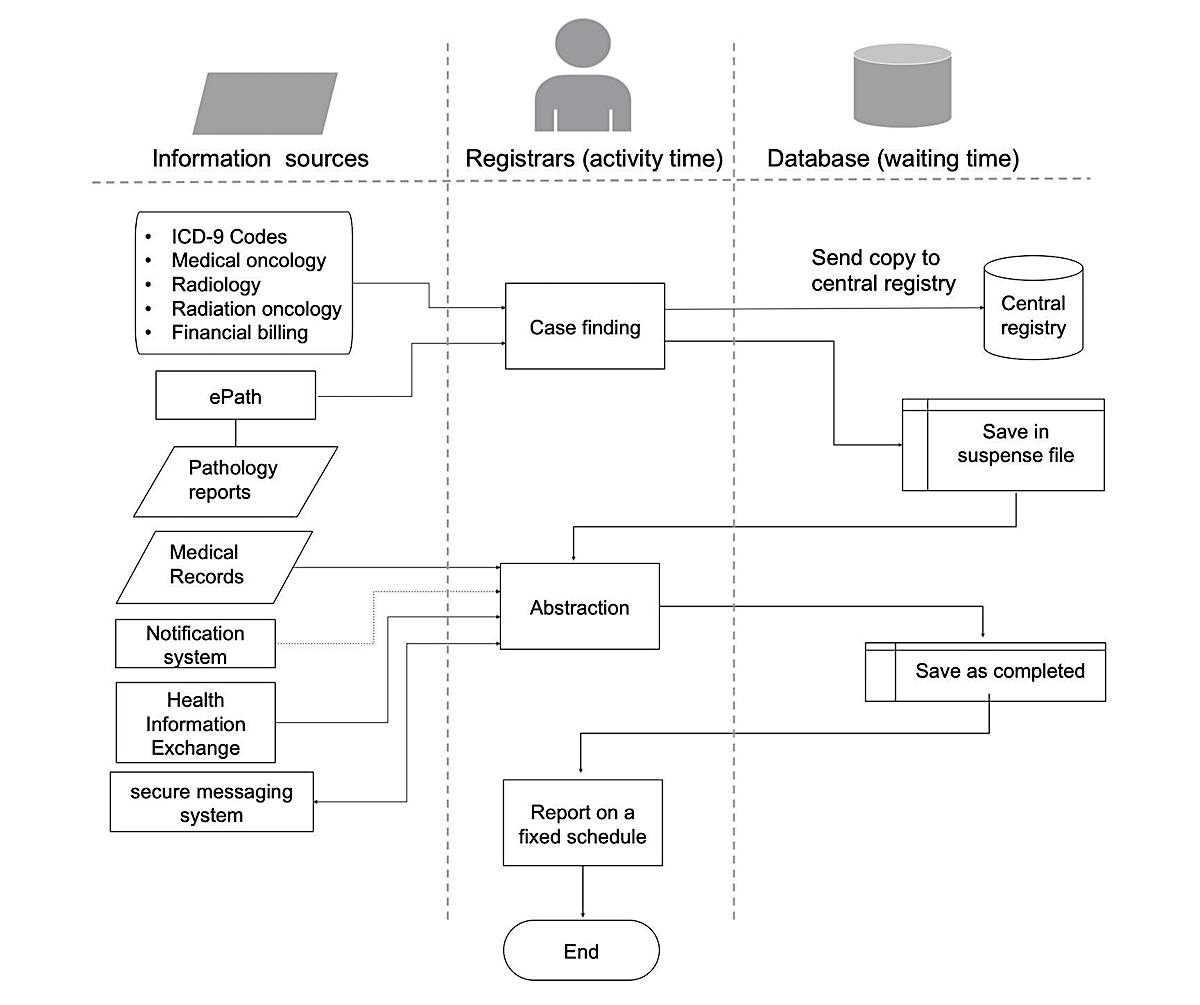
Cancer reporting flowchart for the redesigned workflow. ICD: International Classification of Diseases.

Data from the interviews were utilized to guide the development and refinement of information flow models. We identified sequences of reporting activities, data sources, roles, and the duration of each task. The procedure for the flowchart development followed the hierarchical task analysis technique [29]. The flowchart developed arranges the tasks within the reporting process and the flow of information for both the existing workflow (Figure 1) and the redesigned workflow (Figure 2).

**Figure 3:**
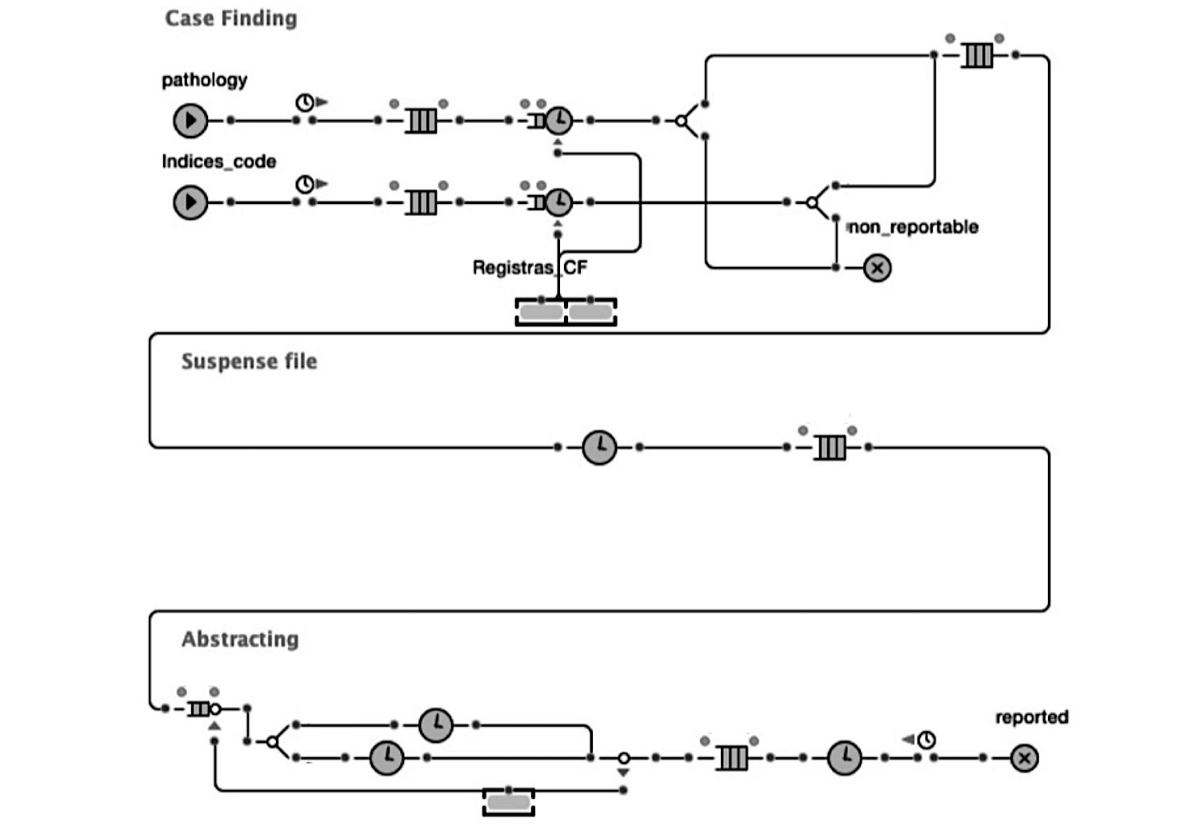
Simulation model for the existing workflow.

Using AnyLogic 7.1, we developed a discrete-events simulation of the current workflow (Figure 3). We used the data collected during the interviews to inform the simulation development (Multimedia Appendix 2). The input data included the duration of activity, waiting time, and number of cases performed. The simulation model provided an indication of the time spent at each phase of reporting (eg, processing time, waiting time, and time cases spend in queue before being processed). The flowchart and simulation model development occurred concurrently with the interviews to test the model’s assumptions and enable iteration. This allowed us to validate the model representation and assumptions with the data obtained from the interviewees.

The simulation model was validated through an iterative process of calibration and comparison with the existing workflow. This validation included ensuring the model represented real-life processes by comparing the total reporting time estimated by the registrars in interviews with the simulation output [30].

**Figure 4:**
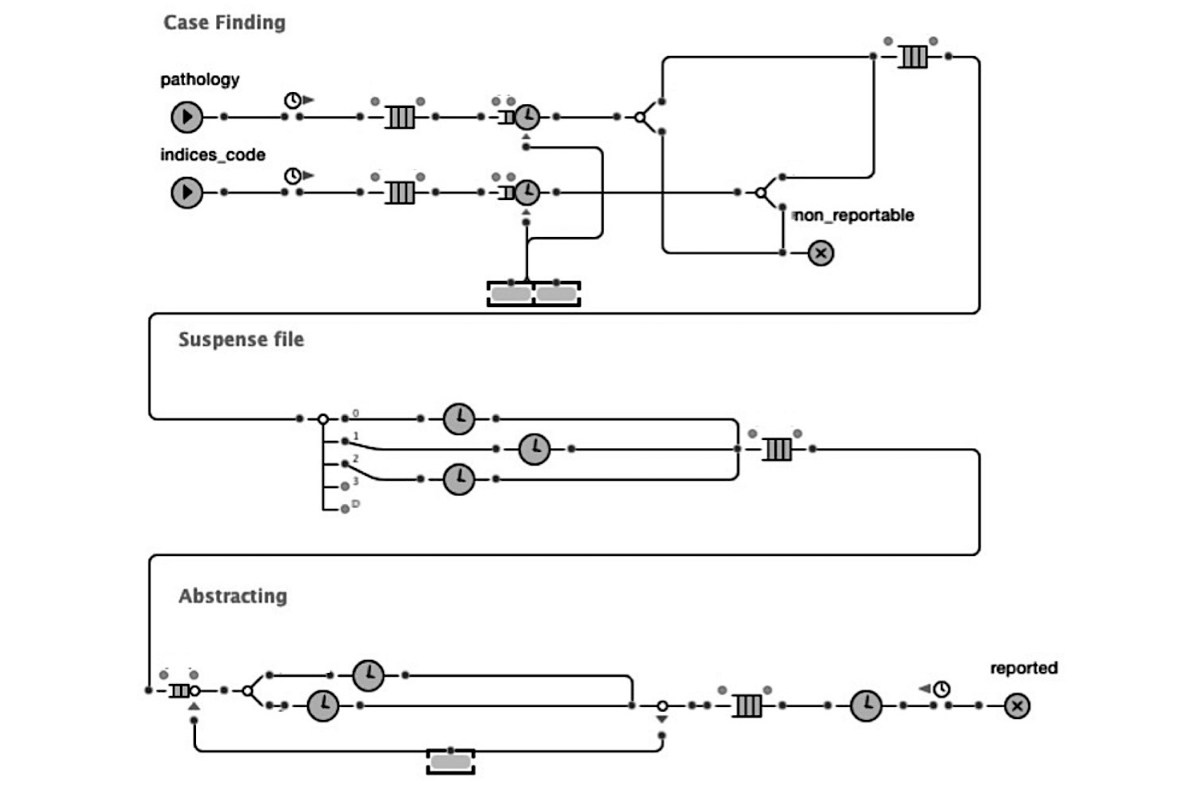
Simulation model for the redesigned workflow.

Thereafter, we simulated the redesigned workflow to estimate the difference in reporting time compared with the current workflow (Figure 4). In the existing workflow, registrars wait about 3-6 months for treatment to be initiated (Textbox 1). To predict the potential savings in reporting time, we needed to estimate the time between diagnoses and receiving treatment, which could not be estimated by the interviewed registrars. Registrars agreed that this time could vary based on factors such as cancer site, cancer stage, and hospital resources. To estimate this time, we used the findings from a previous study by Bilimoria et al that calculate the time between diagnosis and treatment [31]. Bilimoria et al examined 1,228,071 patient records from 1995 to 2005 using data from the National Cancer Database, which represents around 1443 hospitals in the United States. Treatment waiting time was simulated using the (average; minimum-maximum) days for the three cancer types. The calculated values for breast, colorectal, and lung cancer were (24; 14-40), (37; 20-63), and (26; 13-46), respectively [31] (Multimedia Appendix 2). We also used the *Cancer Facts and Figures, 2012* to estimate the proportion of each cancer type at the ISDH cancer registry (ISDH, 2012).

**Estimated time for each reporting step.**

Activity time (tasks performed by registrars)

- Task: Case finding from the pathology reports
  - Time: Daily 1 hour
- Task: Case finding from the International Classification of Diseases-9 list
  - Time: Monthly 1 day
- Task: Abstraction
  - Time: Daily 45 min to 1.5 hours per case

Nonactivity time (waiting time)

- Phase: Suspense file
  - Time: 3-6 months, varies among hospitals

- Phase: Completed cases reside at the local registry before submission
  - Time: An average of 15 days for hospitals with higher caseloads (>300 cases per year)

## Results

### Overview

A total of 14 registrars agreed to participate, and the average interview duration was 28 min (range 17-44 min). Half of the participating registrars reported for larger hospitals (300 beds and over). Out of the 14 registrars interviewed, 6 were reporting for hospitals within Indianapolis and the others were reporting for rural hospitals.

The interview focused on the following areas: (1) understanding the workflow of cancer reporting, (2) estimating the time spent on each phase within the process, and (3) identifying the barriers to rapid reporting. The interview results were organized into the existing workflow description and barriers, recommended workflow, and simulation comparison.

### Existing Workflow Description

Using the interview data, we mapped and described the existing reporting workflow. The reporting process comprises 3 major steps: case finding, abstraction, and reporting (Figure 1). The details of each step are described below.

#### Step 1: Case Finding

When registrars were asked “how does reporting start?,” they reported that the first step is case finding. This involves identifying new cases of cancer that have been diagnosed within a given period. This applies to all inpatients and outpatients diagnosed with or treated for a reportable tumor. Registrars reported that 90% to 95% of the reported cases are identified through pathology reports. Pathology reports are especially useful because they contain detailed information about the cancer diagnosis, histology, and behavior. Some facilities use additional sources for case finding, including hospital admission and discharge records, surgery schedules, cytology reports, oncology reports (medical and radiation), radiology reports, and billing records. Participants suggested that those sources are less informative than pathology reports. Nonetheless, registrars often use multiple sources or refer to medical records to find the information that they need. Data collected during case finding may include demographic information and basic information about the tumor such as site, histology, and behavior. The amount of information collected at this stage is subjected to the information availability and thus, may vary from case to case. Missing information is often completed during the abstracting phase, as the primary goal of case finding is the identification of potentially reportable cases.

Once a case is confirmed as reportable, it is added to a suspense file to await abstraction. In most facilities, case finding is performed daily or weekly (for pathology reports) and monthly (for all other sources). Cases may then reside in the suspense file for several months before abstraction. The rationale for this waiting period is to allow for tests and treatments to be performed and thus, available for inclusion in the report ultimately sent to public health authorities.

#### Step 2: Abstraction

Although case finding provides an initial awareness of a given case, abstraction is more comprehensive and detailed. Abstraction uses different parts of the medical record to collect demographic information, tumor-related information, and information about staging, diagnostic studies, and treatment. When registrars were asked to describe the abstracting process, we found that abstraction is less structured than case finding as registrars flexibly use different parts of the medical record to create a summary.

When registrars were asked, “where in the reporting cycle does the delay exist?,” they reported that abstraction could be delayed when data are not available in local medical records. This is more frequent when patients receive care at an outside facility. Registrars indicated that the percentage of cases that require contacting external facilities varies widely, from 10% to 40%. To access records at outside facilities, reporting registrars often reach out to people at the hospitals where care was provided. These individual contacts may range from health care providers (eg, doctors or nurses) to cancer registrars working at the external facilities. Once all the required information is collected and the abstract is considered complete, it is then saved in preparation for submission.

The interviews revealed that both case finding and abstracting could be performed by the same registrar, especially at smaller hospitals where the number of registrars is limited. Larger hospitals, on the other hand, are more likely to divide the role such that registrars can focus on either case finding or abstracting.

#### Step 3: Submission

Registrars save the completed abstracts and send them in batches to the state registry at fixed time intervals. The submission is made electronically and takes less than 15 min for the entire batch. Facilities with a higher number of cases are required to report abstracts to the state registry at a higher frequency. For example, the ISDH requires hospitals with an average of 1 to 59 cases annually to report their cases once each year; hospitals with an average of 60 to 149 cases annually are required to report their cases quarterly; hospitals with an average of 150 to 299 cases annually are required to report their cases every other month; and hospitals with an average of 300 or more cases annually are required to report them on a monthly basis.

#### Time per Step

We asked registrars to estimate the time it takes to perform each task, and we aggregated the average time estimated (Textbox 1). During interview, we also asked registrars if they encounter a delay or have to wait during the reporting process. The interview results show that the reporting process cycle time contains both activity and waiting times. The activity time includes the time that registrars spend to access and retrieve data, review the records, and enter information into the system. Waiting time, on the other hand, refers to the time during which cases or records reside in the system while no activities are being performed. This includes the time that cases reside in a suspense file before abstraction as well as the time that completed reports reside in the local system before being sent to the appropriate state registry.

#### Existing Workflow Barriers

We aggregated the barriers identified during the interview and grouped them into the following themes. Most reported barriers were related to data exchange, followed by information quality-related barriers (Textbox 2).

#### Data Exchange

The most commonly reported barrier was accessing information at external hospitals. Many of the facilities providing oncology treatment are external or independent. Registrars reported that the percentage of cases that requires contacting external facilities varies from 10% to 40%. While describing the barriers encountered, one interviewee stated:

Getting the information from physicians and letting them know they are not breaking HIPPA if they give us this information. Telling them even if we are not face-to-face with the patients, we are still doing patient care.

**Summary of barriers reported by cancer registrars.**

Theme: Data exchange

- Number of respondents: 8
- Key barriers identified:
  - Difficulty accessing information within facilities outside the hospital network
  - The lack of data exchange between electronic systems

Theme: Information quality

- Number of respondents: 6
- Key barriers identified:
  - International Classification of Diseases codes are not sufficient for confirming the repeatability of the flagged cases
  - Text reports using uncertain language such as “probable,” “suspected,” “likely,” “questionable,” and “possible”
  - Different treating physicians sometimes report contradicting information

Theme: Information processing

- Number of respondents: 5
- Key barriers identified:
  - Combine different events into a single coherent abstract
  - Interpret some of the information in the medical records and translate it to fit the registry requirement
  - Complicated cases with many procedures
  - Large number of nonreportable cases that are flagged to be reviewed

Theme: Administrative tasks

- Number of respondents: 3
- Key barriers identified:
  - Administrative tasks such as reviewing compliancy, serving on the tumor board and on cancer committees
  - Reporting for other institutions with different reporting requirements such as Commission on Cancer

Theme: Technical factors

- Number of respondents: 2
- Key barriers identified:
  - System session timeout
  - Some systems do not have the ability to distinguish the previously reported cases from the new cases

Another interviewee stated:

I tend to go onsite and meet people. I don’t call a lot because some places are not happy giving that information. They want to know who I am and where I am from, so the contact I do have, I build a rapport with and I get the information from them.

When patients receive treatment at an external facility, the abstracting registrar sometimes contacts the registrar working for that facility instead of contacting the physicians or nurses. This is often expressed as a preferable alternative, because they are familiar with the reporting process and requirements. One interviewee also stated that the real obstacle comes with finding out where patients receive treatment, as this is not always indicated in the medical records.

Another information exchange-related barrier was the system inability to exchange information between departments within the same hospital. An example of this would be hospitals that use multiple systems such as legacy systems, paper-based systems, or interoperable systems. One registrar stated that the hospital system does not support some of the technology they wish to use. Because the hospital system is not compatible with the oncology management reporting system, registrars are not able to use all of the features that require data sharing.

#### Information Quality

Registrars reported some issues associated with physician’s notes, such as the lack of information and the ambiguous terminology. Most cancer diagnoses are confirmed through biopsy, but when pathology reports are not available, other information sources such as physician’s notes or diagnostic imaging reports are used. The difficulty arises when uncertain language is used. Terms such as “probable,” “suspected,” “likely,” “questionable,” or “possible” lead registrars to seek more data sources to confirm diagnosis.

A less common barrier identified during abstracting is contradiction in the information found in the records. In some rare cases, registrars find contradictions in the different information sources, such as physician’s notes and pathology reports or even within physician’s notes if multiple physicians treat the same patient.

#### Information Processing

Registrars sometimes expressed some forms of mental load while dealing with information. They collect information from multiple sources and arrange them in chronological order, building a series of events. One interviewee described it as putting together pieces of a puzzle, where they try to find the answer to what they are looking into. The sequence of events has to follow a logical treatment path, using the available data. This process can get complicated when some of the expected events (such as treatment or procedures) are missing. Registrars then will try to find out which data are missing or which procedures were not performed. One registrar commented:

When you do that abstracting for patients you are writing their story, you are the author. You want to make sure you have all the facts, the dates, the treatment collection, date of birth, name, so when you write, your comments have to be clear as to what happened to that patient.

The second factor that contributes to the mental load is the interpretation of the physician’s note. Terminology used may differ from that which is required by the central registries. Interpreting this information requires not only a solid understanding of the domain but also an understanding of the patient’s individual situation and contexts. This can be more challenging for complicated cases with many procedures.

An additional challenge can be presented when using International Classification of Diseases (ICD) codes for case finding. During case finding, registrars search the hospital database for the predetermined set of codes and keywords that may indicate a tumor. This may result in many nonreportable cases also being retrieved by the system. Registrars indicated that only 2.5% to 11% of the cases identified through disease indices are reportable. To filter them, registrars manually review the results to verify their eligibility for reporting.

#### Administrative Tasks

Some registrars indicated that administrative tasks, such as reviewing compliancy and serving on a tumor board and on cancer committees, could be time-consuming. In addition to reporting to state registries, some hospitals voluntarily report to the Commission on Cancer (CoC), which requires continuous follow-up. This involves updating the patient status, cancer status, any recurrence, new cancer, or new treatments. To perform the follow-up, registrars continue searching and updating patients’ information for life. When describing the follow-up required by CoC, one registrar stated:

Going through 3000 plus in the suspense file and only getting 300 or 50 in my case. That is a huge time-consuming part.

#### Technical Factors

Registrars collect information from diverse sources, which requires them to access different systems, paper records, or make phone calls. Being busy with one source will result in inactivity in the previous one, and most electronic systems will log the user out automatically if being inactive for certain period of time. One registrar commented:

My most time-consuming thing for me lately is getting the medical records to work...logging to the system, staying logged in, dealing with connection.

Other barriers were software-related. Some registrars indicated that open source software, such as Rocky Mountain, only provide the basic features and do not provide any of the additional functionalities that can promote an efficient workflow, especially for matching cases and case follow-up.

### Workflow Recommendations

The redesign focused on the deviations that could have the highest impact on the reporting time (Textbox 3). On the basis of the respondents’ feedback, time spent on cancer reporting comprises not only the time spent on tasks but also waiting time, which consumes most of the total reporting time (Textbox 1). Most of this waiting time occurs while patients await treatments and procedures. Respondents further indicated that the time cases reside in suspense files vary between facilities, but the same waiting time is applied to all cases within a given facility. Registrars agreed that procedures and treatments could be performed at different speeds, depending on many factors such as cancer type, cancer stage, and facility resources. This variation suggests that using a standard waiting time for all cases creates an unnecessary delay if treatments are delivered earlier than the anticipated time. We recommend using a notification system (described below) to target this phase of reporting due to its higher impact on timeliness relative to the other phases (Textbox 1). Moreover, cancer registrars reported that data exchange and access to external records was a major barrier during abstraction. On the basis of impact and pervasiveness, we recommend incorporating an electronic pathology reporting system (ePath) for case finding, access to HIE networks, and secure messaging systems (Textbox 3).

**Barriers addressed by recommendations.**

Recommendation: Electronic pathology reporting system

- Barrier theme: Information processing
  - Specific example: Over 90% of the cases identified during case finding are identified through pathology reports

Recommendation: Notification system

- Barrier theme: Not applicable
  - Specific example: Waiting time in the suspense file: cases may take up to 6 months after case finding to abstracting

Recommendation: Access to health information exchange

- Barrier theme: Data exchange
  - Specific example: Difficulty accessing information within facilities outside the hospital network
- Barrier theme: Information processing
  - Specific examples:
    - International Classification of Diseases codes are not sufficient for confirming the repeatability of the flagged cases
    - Text reports using uncertain language such “probable,” “suspected,” “likely,” “questionable,” and “possible”

Recommendation: Messaging system

- Barrier theme: Data exchange
  - Specific example: Difficulty accessing information within facilities outside the hospital network

#### Electronic Pathology Reporting System

About 90% to 95% of cases identified at the case finding stage are identified through pathology reports. Using an ePath system for case finding has been shown to improve reporting timeliness and increase reporting efficiency [32]. Many registrars stated that they had automated the process of case finding from pathology reports and adopted the Public Health Information Network Messaging System for sending Health Level Seven (HL7) messages [32-34].

#### Notification System

We propose adding a notification system between the hospital cancer data management system and the EHR system to notify registrars when new treatments are delivered. Using a notification system would enable registrars to abstract a given case as soon as new treatment data are added to the hospital EHR system instead of the current method, which applies the same waiting time for all cases. Notification systems for workflow optimization have been applied in other health care settings to promote the coordination of care [35]. System notification can be implemented using HL7 Clinical Document Architecture notification messages. Once a new treatment is added to the EHR system, an event can be triggered and the notification system will match it with patient lists in the suspense file. If a match is found, then registrars can be notified about the addition of the new treatment.

#### Access to Health Information Exchange

Indiana hospitals have participated in the Indiana HIE for more than a decade [36,37], yet the exchange does not currently facilitate access for cancer registrars. Utilizing the existing HIE network to facilitate access to information could reduce obstacles to obtaining details about cancer cases and outcomes. Moreover, accessing more information can also improve the accuracy of reporting. Several other states also have an HIE infrastructure that could be similarly utilized. Studies have shown the benefits of HIEs in improving access to clinical data [38-43].

#### Secure Messaging System

Our result shows that registrars encounter difficulties when asking clinicians at external facilities for patient information. As a result, they contact the registrars at the external facilities to access patient information. This relationship was perceived as more conducive to accessing the information needed, given their understanding of each other’s job role and reporting requirements. In this workflow model, we propose the usage of a secure messaging system to facilitate communication among registrars so as to minimize the access barriers to the sharing of information. Studies have shown that the use of secure messaging in other clinical settings improves communication effectiveness among health professionals [44].

**Table 1:** Comparison of existing and proposed methods simulations.

Workflow design		Simulation time	
		1 year	2 years
**Existing workflow**			
	Days (minimum, average, maximum)	102.2, 138.6, 177.8	102.2, 138.6, 180.6
	Percentile (25%, 75%)	128.9, 149.1	128.9, 149.1
	Standard deviation	10.8	10.7
**Recommended workflow**			
	Days (minimum, average, maximum)	19.6, 51.8, 95.2	19.6, 51.8, 95.2
	Percentile (25%, 75%)	39.5, 61.9	39.5, 61.9
	Standard deviation	10.2	9.8
*P* value at 95% CI		.039	<.001

#### Recommended Workflow Steps

Figure 2 shows the flowchart for the redesigned workflow. The steps for the redesigned workflow are as follow:

1. Cancer cases are identified through pathology reports using the ePath system.
2. Registrars review and approve cancer cases identified by ePath.
3. Registrars perform case finding manually for the other data sources.
4. Cases identified as reportable are saved in the suspense file for abstraction. A copy of the identified cases is sent to the state registry and marked as incomplete.
5. EHR sends a notification to the cancer registry management system regarding the delivery of any new cancer-related treatment. If the notification matches any of the cases in the suspense file, then the case will be flagged.
6. The registrar will check the flagged case and start abstracting. If no new treatment is received within 6 months of the date of diagnosis, then the registrar will start abstracting and check the physician’s notes and discharge summaries regarding whether treatment was provided elsewhere.
7. If treatment is received at an outside facility, then registrars will use the HIE to search for external information.
8. If more information is needed, then the reporting registrar can use the secure messaging system to contact other registrars at the outside facility.
9. Registrars save the completed abstracts in the local database to be reported at fixed intervals.

#### Simulation Output

The simulation results show that the redesigned workflow could potentially reduce the reporting time from an average of 138 days to 51 days (Table 1). Although the redesigned workflow added new tasks to minimize some of the barriers identified during interview, most of the reduction in reporting time was attributed to simulating the notification system. Although most tasks take an hour or less, waiting in the suspense file may take up to 6 months (Textbox 1). As seen in our simulation assumption (Multimedia Appendix 2), simulating the notification system enables us to distinguish the time that cases reside in the suspense file among the three cancer types.

## Discussion

### Principal Findings

There is an increasing interest in leveraging cancer registry data to advance the quality of cancer care and bridge the gap between scientific discovery and existing practice [1-4]. Yet, the lengthy reporting time is a major challenge that inhibits the use of cancer registry data for actionable intervention [1-4]. Little is known about the cancer reporting process or the barriers encountered during reporting. In this study, we conducted key informant interviews to understand the details of the reporting process and workflow activities at the hospital level. We examined the time taken at each stage of reporting to target the most time-consuming activities and shorten the reporting process.

Prior research has applied data mining and machine learning techniques to simplify case finding activities and enable automated identification of cancer cases. Although this approach can minimize the time spent on these activities, we found that cancer reporting processes comprise not only active tasks performed by registrars but also inactive waiting times, during which registrars wait for new information about cancer cases to become available in the EHR. These waiting periods occur during the interval of time wherein patients receive diagnostic procedures and treatment. Our findings suggest that the waiting periods can consume more of the total time associated with cancer case reporting than those periods involving active tasks performed by registrars. Consequently, timeliness may be improved by changing the queuing method that is currently applied by registers across hospital types.

Cases generally reside in a suspense file at the hospital for a few months, during which time treatments and procedures are delivered by clinicians and subsequently entered into the hospital’s EHR system. Thus, the first case entered into the suspense file will be the first case abstracted later when the registrar checks for updates. However, procedures and treatments are scheduled and performed at various speeds, depending upon factors such as the cancer type, stage, facility resources [31], as well as other social and clinical patient characteristics. Using a standard waiting time for all cases creates an unnecessary delay if treatments are delivered earlier than anticipated. Adding automated EHR-based notification mechanisms, to inform registrars when new data are available, will enable cancer registrars to abstract case information as soon as it is available instead of waiting a fixed period of time for all cases.

Using EHR-based notification mechanisms could also be applied with two-phase reporting. Two-phase reporting could support the development of “Rapid Learning Systems” [45] where cases can be reported as an incomplete abstract after case finding and updated once treatment and outcome data become available. Technology-enhanced methods will further enable surveillance for timely and high-quality treatment by alerting registrars (or clinicians) when individuals diagnosed with cancer may be overdue for treatment or have been lost to follow-up.

### Limitations

One methodological limitation of our study is the absence of field observations to complement the semistructured interview data, as well as a more quantitative assessment of the prevalence of various barriers through structured surveys. Yet, an advantage of the cancer registrar interviews was the ability to capture rich, in-depth descriptions of a broad range of processes involved in end-to-end cancer reporting. Self-reported interview data are subject to recall bias; however, this threat to validity is lessened by the fact that registrar descriptions generally agreed with one another. We also limited the interviews to experts who could provide insight into the process by focusing on certified registrars who currently report to the ISDH cancer registry. Moreover, we included both large and small hospitals, as well as urban and rural hospitals, to enhance the generalizability of our results.

A second limitation is the method for estimating the simulation input for the redesigned workflow. To conduct the simulation for the redesigned workflow, we needed an estimate of the expected time that cases reside in the suspense file. This is represented by the time from case finding to availability of treatment results. To estimate this time, we used a national study that measured the time from diagnosis to treatment [31]. Although this approach could underestimate the simulation input by disregarding the extra time needed to document the treatment result and add it to the EHR, it could also overestimate the simulation input by disregarding the shortened period for case finding likely to occur with implementation of the ePath system.

Currently, cancer registrars can begin abstraction at the start of the first course of treatment; however, registrars may decide to wait longer to have more complete treatment information to add. For future research, we recommend measuring the time from diagnosis to treatment using the treatment data available at the registry to estimate the minimum reporting time possible for a given rate of completion.

Moreover, our study was limited to cancer registrars within the state of Indiana. Although most state registries have similar reporting requirements and training, we believe evaluating the reporting process in other states will be important to assess the generalizability of our results and recommendations.

### Conclusions

Key barriers to the rapid collection of cancer surveillance information in the existing reporting process include data residing at multiple institutions and the waiting period for the completion of treatment. Our results highlight how health information technologies could be leveraged to overcome these barriers, including ePath systems, HIE, and secure messaging. Understanding the time elapsing at each step within the process helps in prioritizing the needs and estimating the impact of future interventions.

In this study, we discovered that reporting speed cannot be entirely controlled by accelerating the case finding or the abstraction process. Pragmatically speaking, registrars need to wait for treatments and procedures to be performed and entered into the EHR before collecting the data. Appropriate waiting intervals could be better defined by further exploring how much the time from diagnosis to treatment varies for different cancer types. Understanding this variation could help determine the potential value of implementing a notification system, as well as setting reasonable expectations for reporting time by cancer type.

## Appendix

Multimedia Appendix 1 Interview script.

Multimedia Appendix 2 Simulation input.

## Abbreviations

CDC: Centers for Disease Control and Prevention
CoC: Commission on Cancer
CTR: certified tumor registrar
EHR: electronic health record
HIE: health information exchange
HL7: Health Level Seven
ICD: International Classification of Diseases
ISDH: Indiana State Department of Health
NAM: National Academy of Medicine
