# Supplementary material for: Toward Timely Data for Cancer Research: Assessment and Reengineering of the Cancer Reporting Process
Source: JMIR Cancer. 2018 Mar 1;4(1):e4. doi: 10.2196/cancer.7515 (PMC5856936; doi:10.2196/cancer.7515)
Supplement: Multimedia Appendix 1 [file cancer_v4i1e4_app1.pdf]

1. Demographic

- Are you a CTR or a non-CTR?
    - Describe your role in reporting.

2. Workload questions

- Can you estimate the number of cases processed (daily or weekly)?
- How many people in your workplace are involved in reporting?
    - How many of them are CTR and how many are not?
    - Do they perform the same task at the same time?
    - Do they share some of the resources, such as computers or phones? Is it enough?

3. Process workflow questions

- In order, what are the main steps for reporting?
- How does reporting start (what triggers the event to start)?
    - Potential probe: Are there other ways to initiate the process?

This part can be repeated for the main steps: case finding, abstraction, reporting, follow-up.

- Describe the ______ process?
        - Potential Probe: What is the goal of this step?
        - Probes (case finding): Are there cases/cancer types that take longer than others to find/identify? If yes, what are they? Why?
        - Probes (case finding): Do you work on an electronic or paper format?
        - Probes (abstraction): Are there certain types of information that take longer than other to abstract? If yes, how often do you have them?
    - Can you estimate the time it takes you to perform this step (daily or weekly) (range and average)?
    - At which point do you spend most of your time, and why?
    - Can you estimate the time you spend on this part?

Repeat for abstraction, reporting, follow-up.

- How do you know when a process is complete?
    - Are there any other possible outcomes? If yes, please describe?

4. Efficiency Questions

- How often you encounter a delay or have to wait during the reporting process? If often, then:

Potential probes:

    - Where in the reporting cycle does the delay exist?
    - What could happen?
    - Why would it happen?
    - How bad is it?
    - And when it happens, what you do about it? Is it effective?

- Are there any parts of the reporting process you wish you could eliminate or fix? If yes,
    - Which part is it?
- Why? Is there a time when you have to start over or repeat work? If yes,
    - How often? And, if often,
        - When (at what part), and why?

5. Other

- Regardless of the current constraints and limitations, what would the ideal reporting system look like?
- Any other comments you would like to add?
