# Supplementary material for: Toward Timely Data for Cancer Research: Assessment and Reengineering of the Cancer Reporting Process
Source: JMIR Cancer. 2018 Mar 1;4(1):e4. doi: 10.2196/cancer.7515 (PMC5856936; doi:10.2196/cancer.7515)
Supplement: Multimedia Appendix 2 [file cancer_v4i1e4_app2.pdf]

Case loads for case finding per CTR:

Cases from ICD codes:

Monthly: 109 cases

0.9% of the ICD codes reviewed during case finding make it to the abstracting phase

From pathology reports

40 reports a week

50% of the pathology reports reviewed during case finding make it to the abstracting phase

Received weekly and reviewed daily (existing workflow)

Received daily and reviewed daily (redesigned workflow)

Abstracting capacity per CTR:

Daily: five abstracts (four if needs external sources)

20% of the cases being abstracted will require searching for information at external hospitals or facilities.

Suspense file: existing workflow (min, avg, max)

(65, 85, 110) (min, avg, max) working days for all cases

Suspense file: redesigned workflow (min, avg, max)

(10, 17, 28) working days for breast cancer

Accounts for 33% of the total cases being simulated

(14.2, 26.4, 45) working days for colorectal cancer

Accounts for 41% of the total cases being simulated

(9.5, 18.5, 32.8) working days for lung cancer

Accounts for 26% of the total cases being simulated

Saving before submission (min, avg, max)

(0, 10, 20) working days
